# Identification and Characterization of Genes Involved in Ecdysteroid Esterification Pathway Contributing to the High 20-Hydroxyecdysone Resistance of *Helicoverpa armigera*

**DOI:** 10.3389/fphys.2020.00508

**Published:** 2020-06-09

**Authors:** Hengtong Duan, Xin Yang, Zhanyao Bu, Xinyue Li, Ze Zhang, Wei Sun

**Affiliations:** Laboratory of Evolutionary and Functional Genomics, School of Life Sciences, Chongqing University, Chongqing, China

**Keywords:** phytoecdysteroids, detoxification, ecdysteroid esterification pathway, transcriptome, cotton bollworm

## Abstract

20-Hydroxyecdysone (20E), the most important regulator for insect development, is also a major component in phytoecdysteroids in plants. Therefore, this plant-derived hormone is considered as a potential natural product for use in pest management. However, some insects show high resistance to it, and the molecular mechanism of their resistance is still unclear. In this study, we find that the cotton bollworm *Helicoverpa armigera* larvae show high tolerance to artificial foods containing up to 50 μg 20E without any detrimental effects on growth and development. High performance liquid chromatography analysis indicates that high efficiency to transform the ingested 20E through an ecdysteroid esterification pathway may contribute to the resistance. Furthermore, comparative transcriptome analysis of the larvae’s midgut after 20E treatment identifies two genes (long-chain-fatty-acid–CoA ligase, Long-FACL; sterol O-acyltransferase, SATF) involved in the pathway. Transcriptome and real-time PCR show the Long-FACL gene can be significantly induced by 20E, and this induction is only detected in the midgut. However, 20E has no effect on the transcript of the SATF gene. Moreover, the heterologously expressed protein of the SATF gene shows the ecdysteroid-22-O-acyltransferase activity that requires fatty acyl-CoA, which is produced by Long-FACL. Taken together, our results identify and demonstrate the genes involved in the ecdysteroid esterification pathway conferring high resistance to 20E in the cotton bollworm, *H. armigera*.

## Introduction

Ecdysteroids are one of the most important hormones in insects. They are recognized as essential regulators controlling molting, metamorphosis, and reproduction in insects. Since the isolation of ecdysone from silkworm pupae in 1954, ecdysteroid analogs called phytoecdysteroids were also discovered in plant species (Butenandt and Karlson, 1954; Nakanishi et al., 1966; Lafont and Horn, 1989; Dinan, 2001). Many plants contain a relatively high concentration of ecdysteroids (up to 3% of their dry weight) (Dinan, 2001). Moreover, the major form of phytoecdysteroids in plants is 20-hydroxyecdysone (20E), which is also the most biologically active ecdysteroid in insects (Lafont and Horn, 1989). Therefore, phytoecdysteroids are considered to be a defensive system to protect plants against phytophagous invertebrates (Adler and Grebenok, 1999; Mithöfer and Boland, 2012). In this regard, phytoecdysteroids were indeed demonstrated to be antifeedants toward some insects, such as the dark-winged fungus gnat (*Bradysia impatiens*) and polyphagous Japanese beetle *Popillia japonica* (Schmelz et al., 2002; Jurenka et al., 2017). More importantly, phytoecdysteroids could execute direct toxicity to insects. Orally ingested low levels of phytoecdysteroids could disrupt normal development, reduce fecundity, or induce death of some lepidopteran insects, such as the sweet-potato whitefly *Bemisia tabaci* and the persea mite *Oligonychus perseae* (Kubo et al., 1981, 1983; Tanaka and Naya, 1995; Blackford and Dinan, 1997a, b; Mondy et al., 1997). Consequently, phytoecdysteroids have been suggested to be a good candidate for pest management (Soriano et al., 2004; Aly et al., 2011; Jurenka et al., 2017; Chaubey, 2018).

However, previous studies have reported that a number of pests, especially certain noctuid insects, showed high resistance to phytoecdysteroids. Some *Helicoverpa* species (*Helicoverpa virescens* and *Helicoverpa zea*) can tolerate high concentrations of 20E in their diet without any detrimental effects (Kubo et al., 1981). Blackford et al. (1996, 1997) also found that exogenous application of 20E could not affect the normal growth and development of *Spodoptera littoralis* and *Lacanobia oleracea* (Blackford et al., 1996; Blackford and Dinan, 1997b). Furthermore, in those noctuid insects, the high resistance is mainly due to the effective conversion of the ingested ecdysteroids into 22-long-chain-fatty-acyl esters in the gut (Kubo et al., 1987; Robinson et al., 1987; Zhang and Kubo, 1993; Blackford et al., 1997). The ecdysteroid-22-acyl esters show over 100 times less activity than 20E (Zhang and Kubo, 1992b). In noctuid insects, the conversion is considered to be an important pathway of detoxification of ingested exogenous ecdysteroids. This esterification process is mediated by ecdysteroid-22-*O*-acyltransferase, which biochemical characteristics have been described in previous studies (Zhang and Kubo, 1992a, b; Kubo et al., 1994). However, the gene encoding ecdysteroid-22-*O*-acyltransferase is still unclear.

The cotton bollworm, *Helicoverpa armigera* (Lepidoptera: Noctuidae), is the most significant and impactful pest in agriculture. The pest is a typical polyphagous insect, which feeds on at least 180 plant species from >30 families, causing serious crops damage and economic losses (Martin et al., 2005). The wide feeding spectrum of the cotton bollworm indicates that some efficient processes must be involved in detoxifying the plant-derived substances. Similar to other noctuid insects, the cotton bollworm can also show resistance to 20E through the ecdysteroids esterification pathway (Robinson et al., 1987). In this study, we used the cotton bollworm as a model, and performed transcriptomic analysis to measure the effect of exogenous 20E on *H. armigera*. Thereafter, we identified and characterized the function of the genes involved in the ecdysteroids esterification pathway. The data shown in this study will help us to understand how insects adapt to and conquer the secondary metabolites of their host plants.

## Materials and Methods

### Insects Culture

Eggs of *H. armigera* and artificial diets were purchased from Keyun Bioinsecticide Research and Development Center of the Chinese Academy of Sciences Institute of Zoology (Henan, China). The larvae were reared on an artificial diet at 27°C under a 12/12-h light/dark photoperiod.

### Insect Treatment

The artificial diet was cut into small pieces of diet blocks (about 25 mg/block), and then the blocks were mixed with different amount of 20E (20 μg/block; 50 μg/block; Sigma-Aldrich, St. Louis, MO). The same size diet blocks with 20% ethanol were used as the control. For ingestion experiments, the day 1 larvae of the sixth instar cotton bollworm were firstly starved for 6 h to accelerate feeding rate, and then each larva was fed on one mixed diet block. All the larvae could completely eat the diet block within 1 h. Then the larva was transferred to a normal diet block (about 0.5 g/block). Larvae and feces were weighted at different time points after treatment. In addition, the remaining foods at each measured time point were also weighted, and then were replaced by a new known weight of an artificial diet block.

According to previous studies, we calculated some nutritional parameters to measure the effects of ingested 20E on larvae: weight gain, amount of ingested food, digestibility, efficiency of conversion of digested food (ECD), and efficiency of conversion of ingested food (ECI). The calculation formulas of those parameters are the same as in the previous study (Blackford et al., 1996).

### Dissection and Extraction

For each time point, a hemolymph of each larva was collected by bleeding the proleg. Methanol was added to the hemolymph (100 μL) from the larva to a final volume of 1 mL. The feces and fat body dissected from each larva were extracted overnight with 2 mL of methanol. The supernatant was collected and evaporated. The dried samples were dissolved in ethanol and stored at −20°C.

### Total RNA Isolation and RNA-seq

After 20E or 20% ethanol treatment at different time points, the midgut, head, fatbody, and epidermis were dissected on ice and immediately frozen and stored in liquid nitrogen, respectively. Every tissue sample was collected from five larvae. Previous studies have shown that the larval midgut was the main tissue to detoxify the exogenous 20E. Therefore, the midgut was collected at 3 h after hormone treatment to perform transcriptomic analysis.

All dissected tissues were grinded in liquid nitrogen to powders. Total RNA was extracted by the Ultrapure RNA kit (Beijing CoWin Biotech, Beijing, China) and treated with DNase I (Takara Bio, Shiga, Japan) to remove the genomic DNA contamination. The RNA was quantified by the UV spectrophotometer.

For transcriptome sequencing, the RNA samples were sent to the Biomarker Technologies Corporation (Beijing, China) for cDNA library construction and RNA-seq by Illumina HiSeq X Ten (Illumina, San Diego, CA, United States) with 125 bp paired-end reads according to the manufacturer’s instructions. The raw data of RNA-seq has been submitted to NCBI (SRA accession: PRJNA588578).

### Analyses on RNA-seq Data

The transcriptomic reads data were mapped to the *H. armigera* reference genome (GenBank assembly accession: GCA_002156985.1) using HISAT2 software and analyzed using StringTie (Pertea et al., 2016). HTSEQ software was used to calculate read counts of each gene (Anders et al., 2015). The expression levels of genes were estimated using TBTools and normalized using the TPM (Transcripts per million; Chen et al., 2018). DEseq2 R package was performed to identify differently expressed genes (DEGs), and genes with Log2-fold-expression-change greater than 2, and corrected *P*-values less than 0.05 were considered to be differentially expressed (Love et al., 2014).

Gene ontology term (GO)^1^ annotations were assigned by Blast2GO software (b2g4pipe_v2.5; Götz et al., 2008). The KEGG (Kyoto Encyclopedia of Genes and Genomes) annotations were performed using Blastall software against the KEGG database^2^ (Kanehisa and Goto, 2000). KEGG and GO enrichment analyses of DEGs were performed on an online platform^3^.

### Real-Time PCR

For each treatment shown above, 1 μg RNA was reverse transcribed to the first strand of cDNA by the EasyScript one-step gDNA removal and cDNA synthesis SuperMix kit (TransGen Biotech). The specific primers were designed and used in the quantitative real-time PCR analysis (Supplementary Table S1). The quantitative real-time PCR was performed using a real-time PCR detection system (CFX96, Bio-Rad, Hercules, CA) with a QuantiNova SYBR Green PCR Kit (Qiagen). The PCR was carried out as follows: 2 min at 95°C, followed by 40 cycles of 5 s at 95°C and 15 s at 60°C. The cotton bollworm ribosomal protein L3 gene (GenBank ID no. XM_021328338) was used as the reference gene.

### Eukaryotic Expression of the Candidate Genes

The cDNA of the candidate genes was cloned into the pMK33/pMtHy-based vector between the metallothionein promoter (pMT) and the SV40 polyadenylation. The constructed plasmid was confirmed by sequencing. Then the recombinant plasmids were transfected into a *Drosophila S2* cell line using an effectene transfection reagent (Qiagen). The transfected method was used according to the manufacturer’s instructions. Twenty-four hours after transfection, a final concentration of 500 μM copper sulfate was added to induce the expression of the targeted genes for 18 h. Then the cells were homogenized in 50 mM Tris–HCl (pH 7.4) containing 1 mM EDTA. The protein was quantified using the BCA protein assay.

To examine whether the gene was expressed, western blotting analysis was performed. The Flag antibody (at a dilution of 1:5000; Beyotime, Shanghai, China) was used to detect the recombinant protein. The procedure of the western blotting was described previously (Sun et al., 2012).

The DNA sequences of candidate genes obtained in this study were deposited in GenBank (Genbank ID, SATF: MN688744; Cyp6B2: MN688745; Long-FACL: MN688746).

### Enzyme Assay, Ecdysteroid Extraction, and High Performance Liquid Chromatography Analysis

To measure the enzyme activity, 500 μL reaction, containing 0.1 mg crude recombinant enzyme solution, 100 μg 20E, 0.06 mM oleoyl-CoA which served as acyl-group donor, and 10 mM phosphate buffer (PH 7.0), was incubated for 30 min at 20°C (Kubo et al., 1994). The reaction was quenched with 4.5 ml ice-cold 100% methanol, and proteins were removed by centrifugation. The supernatant was collected and evaporated. The dried samples were dissolved in ethanol and stored at −20°C. Ecdysteroids were analyzed by reversed-phase high performance liquid chromatography (HPLC) using a C18 Nova-Pak cartridge (4.6 × 250 mm; Waters Associates) which was performed on a Hitachi Primaide HPLC system (Hitachi, Japan) linked to a Dual λ Absorbance UV detector set at 245 nm. The mobile phase consisted of methanol-water (93:7, v/v), and the elution rate was set at 1 mL/min.

In addition, the concentration of 20E and its derivates was calculated by comparing the peak area of the analyte in the sample with the peak area of the standard of a known concentration.

### Mass Spectrum

Mass spectrometry was performed to identify the metabolites of 20E. Mass spectrometry was conducted by a Finnigan TSQ Quantum Ultra AM spectrometer (Thermo Electron Corp., San Jose, CA) equipped with an ESI source in the positive ion mode. The spray voltage was fixed at 3.0 kV and the capillary temperature at 200°C. The collision energy was fixed at 20 eV. Nitrogen was used as the sheath (20 psi) and auxiliary (8 psi) gas.

### Statistical Analysis

All the experiments shown above were independently repeated three times. All statistical analyses in this study were performed in the statistical R package (Student’s *t* test; Mann-Whitney *U* test).

## Results

### Ingested 20E Has No Effect on the Growth and Development of the Cotton Bollworm

**FIGURE 1:**
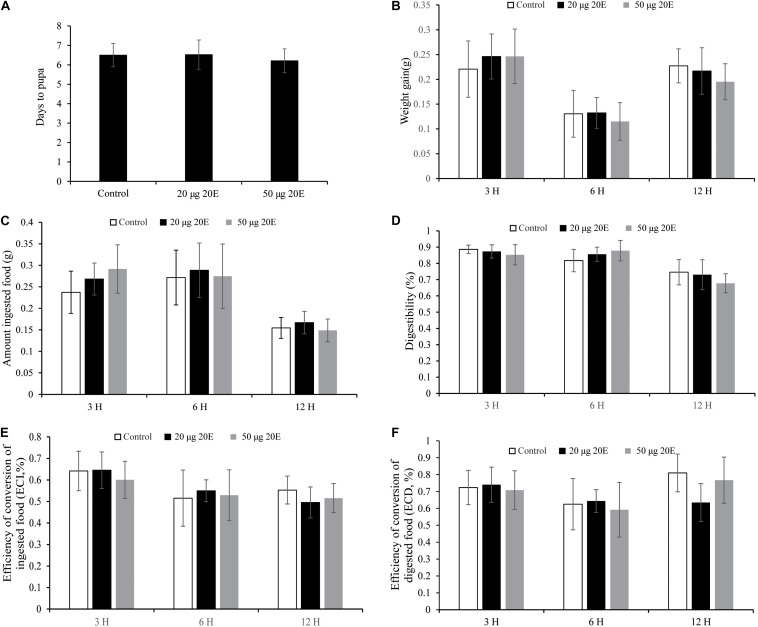
Effects of the ingested 20E on the growth and development of the cotton bollworm larvae. **(A)** The time to pupation of the larvae reared on food treated with control or different concentrations of 20E. **(B)** The weight gain of the larvae reared on food treated with control or 20E. **(C)** The average amount of ingested food by the control or 20E treated larvae. **(D)** The digestibility of the larvae treated with control or 20E; **(E)** The efficiency of conversion of ingested food (ECI) of the larvae treated with control or 20E. **(F)** The efficiency of conversion of digested food (ECD) of the larvae treated with control or 20E. Mann–Whitney *U* test was used to do the statistic analysis.

To ascertain whether exogenous 20E may affect growth and development, we mixed 20E with artificial diet and fed the 1-day-old cotton bollworm larvae. Here, we utilized two different concentrations of 20E (20 μg/larva and 50 μg/larva) which are almost 15 and 36 folds higher than the top peak of 20E titer during the metamorphosis stage, respectively (Kang et al., 2019). The artificial diet containing high concentrations of 20E (up to 50 μg/larva) were applied. All the tested larvae could quickly consume the treated diet within 1 h and then were fed on a normal artificial diet. We found the high dosage of the hormone did not affect the growth and development of cotton bollworm larvae. The larvae treated with 20E gained weight and pupated at the same time as the controls, producing normal pupae with a similar weight and emerged as perfect adults (Figures 1A,B). Meanwhile, we also measured some nutrition parameters to further assess the influence of the ingested 20E. Larvae treated with 20E could consume a similar amount of artificial diet to the control (Figures 1C,D), and the conversion of ingested (ECI) and digested (ECD) food into the body also had no significant difference among the three tested groups (Figures 1E,F). Thus, the cotton bollworm larvae has a high tolerance to the exogenous ingested 20E.

**FIGURE 2:**
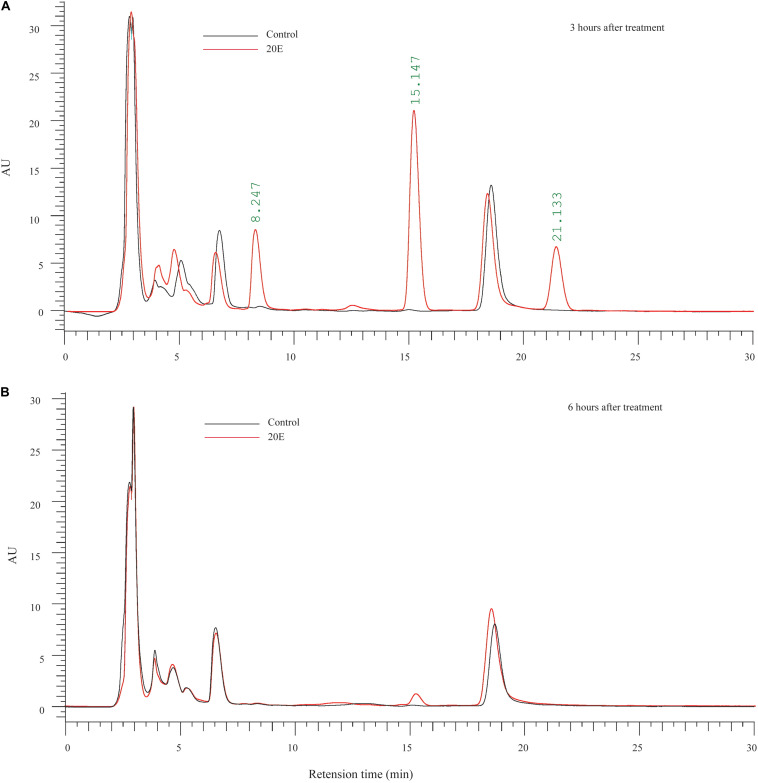
Metabolic fate of ingested 20E. **(A)** HPLC analysis of ecdysteroids extracted from the feces of larvae at 3 h after control or 20E treatment. **(B)** HPLC analysis of ecdysteroids extracted from the feces of larvae at 6 h after control or 20E treatment. Black lines represent the extraction from control treated larvae, and red lines represent the extraction from 20E treated larvae.

To examine the metabolic fate of ingested 20E in larvae of the cotton bollworm, feces was collected from larvae fed on 20E (20 μg/larva) after treatment. Three hours after treatment, compared with the control, we found three additional peaks in the feces of the 20E fed larvae. According to the elution time of the standard sample, the first additional peak (FAP, retention time: about 8 min) is 20E (Figure 2A). Furthermore, we used mass spectrum to identify the second additional peak (SAP, retention time: about 15 min) and the third additional peak (TAP, retention time: about 21 min). Significant fragmentation peaks of the SAP were observed at *m/z* 745, 727, 709, 463, 445, 427, and 301. For the TAP, the main ion peaks were at *m/z* 747, 729, 711, 463, 445, 427, and 301 (Supplementary Figure S1). According to the mass spectra shown in previous analysis, the SAP and TAP are the 20E-22-oleate and 20E-22-stearate, respectively (Kubo et al., 1987). In addition, the amount of the 20E and metabolites from feces of each larva is calculated. At 3 h after hormone treatment, 3.834 ± 0.145 μg 20E, 8.810 ± 0.553 μg 20E-22-oleate, and 2.875 ± 0.245 μg 20E-22-stearate were detected. This result indicated about 72.34% ingested 20E were excreted in feces in the form of 20E (19.17%) and 22-acyl esters (58.43%). Moreover, 6 h after treatment, 20E and 20E-22-stearate peaks disappeared, and only a small peak of 20E-22-oleate could be detected (Figure 2B). We also detected minor amounts of 20E-22-oleate and 20E-22-stearate in the hemolymph and fat body (Supplementary Figure S2), indicating ecdysteroid 22-acyl esters can pass through the gut and store in other tissues.

Taken together, our results indicated that the high resistance to 20E is contributed to by the transformation into lipophilic ecdysteroid esters.

### Transcriptomic Analysis

**FIGURE 3:**
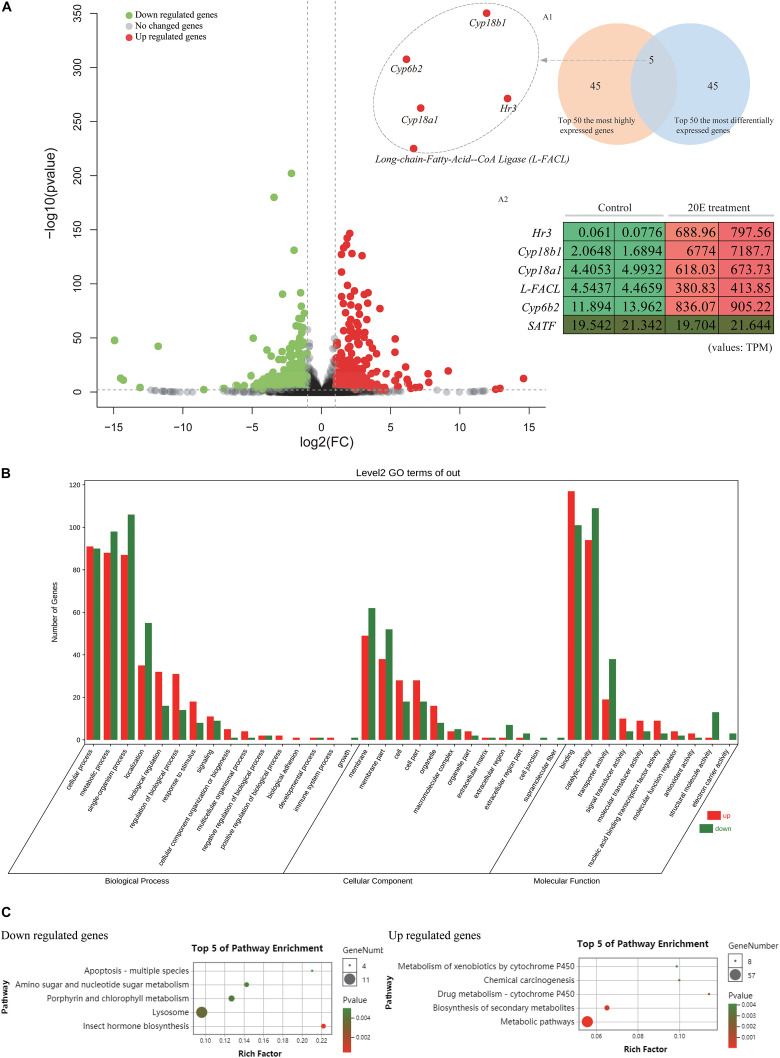
Comparative transcriptome analysis of the larvae midgut after control and 20E treatment. **(A)** Volcano plot of the fold change of transcripts in 20E treated larvae midgut compared to control; Inner figure A1 represents Venn diagram between top 50 most differentially expressed genes and top 50 most highly expressed genes in 20E treatment sample; Inner figure A2 represents the TPM value of the candidate genes identified by Venn diagram; TPM means transcripts per million. **(B)** Gene ontology analysis of the differently expressed genes between control and 20E treated sample. **(C)** KEGG analysis of the differently expressed genes between control and 20E treated sample.

As shown above, exogenous 20E has no effect on the normal growth and development of the *H. armigera* larva. We further examined whether 20E, an important developmental regulator, had roles of gene expression alteration at the transcriptomic level. Firstly, we identified 930 differently expressed genes (DEG) by comparing the gene expression levels between control and 20E treated larval midgut. Among them, 448 genes were significantly up-regulated by 20E, and 482 genes were decreased (Figure 3A). Compared with the down-regulated genes, the functional GO terms of up-regulated genes were mainly enriched in molecular regulation and signal transduction (Figure 3B). As the important steroid hormone, 20E could elicit its signal transduction and affect the expression change of several genes. In the up-regulated gene dataset, we identified 15 transcription factors. Almost half of those TFs (7/15) are known 20E response genes, such as ecdysone receptor (EcR), broad-complex, and E75 (Supplementary Table S2). Among them, hormone receptor 3 (HR3), which is considered as a central regulator in 20E-driven developmental transitions, showed the largest expressional change after 20E treatment (Lam et al., 1997). Furthermore, KEGG analysis was performed. Down regulated genes were enriched in insect hormone biosynthesis, lysosome, and metabolism (Figure 3C). For the up-regulated genes, the metabolic pathway, biosynthesis of secondary metabolites, and some detoxing related cytochrome P450 were enriched in KEGG analysis, indicating the cotton bollworm may promote its detoxing system to conquer the exogenous 20E.

In addition, the feeding test shown above indicates that 20E has no detrimental effects on the growth and nutrition conversion of the larvae. Therefore, we checked the expression of several digestive enzyme genes. The main digestive enzymes of insects are carbohydrases (β-glucosidase, α-amylase, trehalase, α-glucosidase, and β-galactosidase), lipases, and proteinases (trypsin and aminopeptidase) (Terra and Ferreira, 2012). From midgut transcriptomic data, we identified several genes (Table 1). Compared with control, the transcript level of most of the digestive enzyme genes were not significantly changed after 20E treatment (Log2(fold change) > 2). This result further supports that the cotton bollworm can tolerate high concentrations of 20E without altering its digestion ability.

**TABLE 1:** Identification and expression of genes encoding digestive enzymes.

Classification	Gene ID	AVG. TPM-Control	AVG. TPM-20E	log2(fc)	FDR	Annotation
Carbohydrases	MSTRG.11222	603.015	677.625	0.168293	0.175097	α-Glucosidase
	MSTRG.1618	36.655	45.505	0.312015	0.58751	α-Glucosidase
	MSTRG.9541	59.56	57.915	–0.04041	1	α-Glucosidase
	MSTRG.8235	20.44	20.67	0.016143	1	α-Glucosidase
	MSTRG.11087	558.86	387.52	–0.52822	1.29*E*−08	α-Amylase
	MSTRG.11086	1737.805	1669.605	–0.05776	0.903124	α-Amylase
	MSTRG.9468	2387.025	2526.855	0.082129	0.4770584	α-Amylase
	MSTRG.9467	78.925	80.155	0.02231	1	α-Amylase
	MSTRG.990	44.175	33.755	–0.38813	0.4591	Trehalase
	MSTRG.2686	3.885	5.2	0.420597	1	β-Galactosidase
Lipases	MSTRG.1592	2373.865	2119.01	–0.16385	0.0704606	Lipase
	MSTRG.1587	32.05	30.34	–0.0791	1	Lipase
	MSTRG.1594	29.765	4.79	–2.63552	1.82*E*−08	Lipase
Proteinases	MSTRG.1715	22.44	11.905	–0.91451	0.089117	Tyrpsin
	MSTRG.10422	1037.475	827.915	–0.32552	0.0006072	Aminopeptidase N
	MSTRG.10425	1095.79	1414.215	0.36803	1.84*E*−05	Aminopeptidase N
	MSTRG.10428	1124.28	735.375	–0.61245	4.52*E*−13	Membrane alanyl aminopeptidase
	MSTRG.10429	788.08	619.355	–0.34758	0.0001199	Membrane alanyl aminopeptidase

### Identification and Expression of Candidate Genes

Previous studies showed that the expression of ecdysone degradation enzymes, such as ecdysone oxidase and Cyp18a1, could be induced by ecdysteroids (Li et al., 2014; Sun et al., 2017). Therefore, we focused on the 20E up-regulated genes from transcriptome data to identify the candidate enzymes contributing to the high ecdysteroid resistance of the cotton bollworm. Firstly, we used the Venn diagram between the top 50 most differentially expressed genes and top 50 most highly expressed genes in 20E treatment sample to filter the target genes (Figures 3A1,A2). Five genes were detected in the intersection of the two datasets. Interestingly, four of five genes were annotated as enzymes including three cytochrome P450 genes (*cyp6b2, cyp18a1*, and *cyp18b1*) and a long-chain-fatty-acid–CoA ligase (Long-FACL). Among them, the function of one cytochrome P450 gene annotated as cyp6b2 is still unknown. Cyp18a1 is served as 26-hydroxylase and mediates the inactivation of the ecdysteroids (Rewitz et al., 2010; Guittard et al., 2011). Cyp18a1 has been identified in different insects, while as its paralog gene – cyp18b1 is only reported in *Bombyx mori* (Li et al., 2014). To trace the evolution of cyp18 genes, we attempted to search for more cyp18 genes in other insects. As a result, cyp18a1 was widely distributed in different insects including Lepidoptera, Diptera, Coleoptera, and Hymenoptera. Interestingly, cyp18b1 was only detected in lepidopteran insects. Furthermore, phylogeny analysis showed cyp18a1 proteins from different species were clustered together, and cyp18b1 proteins formed a single clade (Supplementary Figure S3). This result indicates cyp18a1 underwent lineage specific gene duplication after the Lepidoptera-other taxa split generating its paralog-cyp18b1.

For the Long-FACL, the enzyme plays key roles in various metabolic and regulatory processes by catalyzing the formation of fatty acyl-CoA (Hisanaga et al., 2004). More importantly, fatty acyl-CoA was reported as the co-substrates (acyl-group donor) for the ecdysteroid-22-*O*-acyltransferase activity (Kubo et al., 1987, 1994; Zhang and Kubo, 1992a, b). Therefore, we thought Long-FACL may take part in the ecdysteroids esterification pathway.

Ecdysteroid-22-*O*-acyltransferase belongs to the family of membrane-bound *O*-acyltransferase (MBOAT; Zhang and Kubo, 1992a, b; Kubo et al., 1994). From the transcriptome data, only four MBOAT genes had a transcription signal, and none of them were differently expressed between control and 20E treatment (Supplementary Table S3). In addition, according to previous studies, ecdysteroid-22-*O*-acyltransferase is located on the plasma membrane of the gut epithelial cells (Kubo et al., 1994). Therefore, based on the subcellular localization, transcript level, and annotation, MSTRG.8235 (Sterol *O*-acyltransferase, SATF) was considered as potential candidate of the ecdysteroid-22-*O*-acyltransferase (Supplementary Table S3).

**FIGURE 4:**
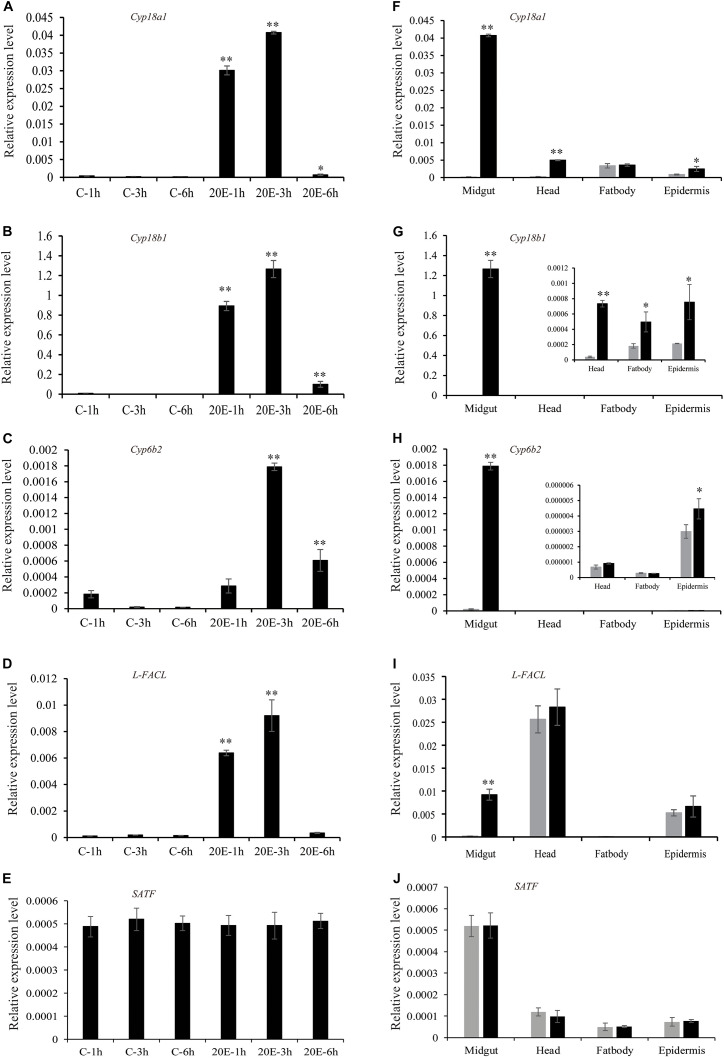
Expression pattern of the candidate genes after 20E treatment. **(A–E)** The expression of the candidate genes in cotton bollworm larvae midgut at different time points after control or 20E treatment. **(F–J)** The expression of the candidate genes in different tissues at 3 h after control or 20E treatment. *p value via student’s *t*-test based on three replicates (*p < 0.05; **p < 0.01).

In addition, we performed real-time PCR to confirm the RNA-seq data and surveyed the expression pattern of the genes identified above. The expression pattern of those genes by qRT-PCR was consistent with the transcriptomic changes of RNA-seq data (Figure 4). In order to further examine the effect of 20E on gene expression, more time points after the hormone treatment were chosen. All genes, excluding SATF, could be quickly induced by 20E from 1 h after treatment, and reached expression peaks at 3 h after treatment. Moreover, for three cytochrome P450 genes, the induction mediated by 20E was detected in different tissues, such as head, fat body, and epidermis. However, Long-FACL was only induced in the midgut. Meanwhile, we surveyed the 5′ upstream of the Long-FACL gene and identified several 20E related cis-regulatory sites by bioinformatics prediction^4^, such as EcR/ultraspiracle (USP), broad-complex (Br-C), and E74, further indicating it is a 20E inducible gene (Supplementary Figure S4). For SATF gene, the gene showed the constant and obvious expression in the midgut, and steroid hormone had no effect on its transcript.

### Heterologous Expression and Enzyme Assay

**FIGURE 5:**
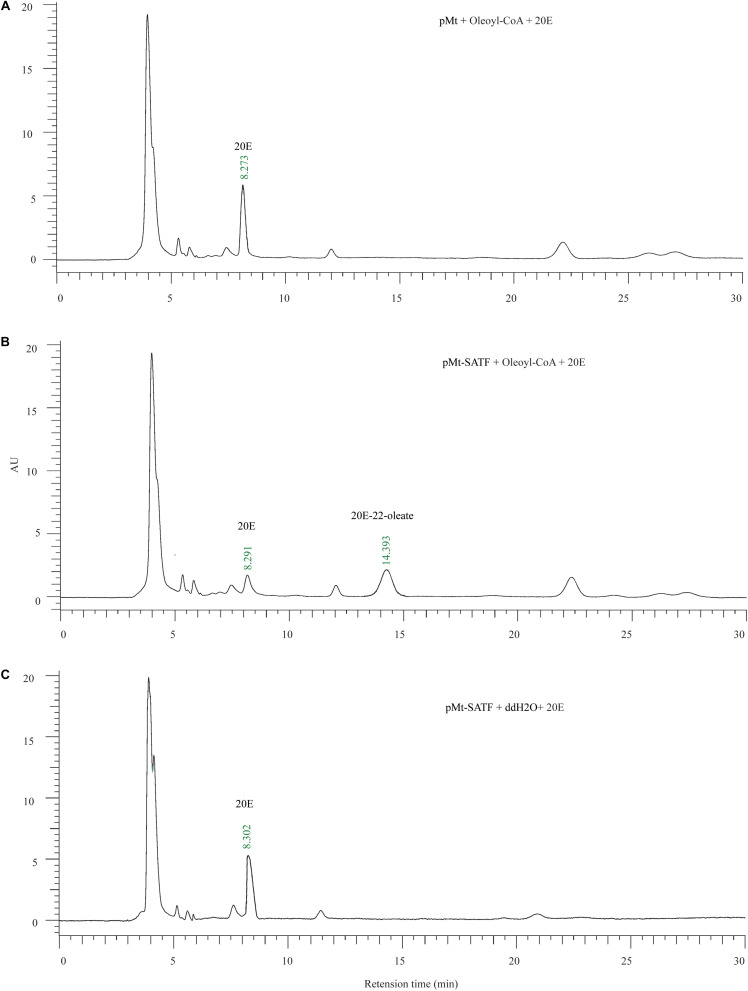
Enzymic activity of recombinant Sterol O-acyltransferase, SATF proteins. **(A)** The enzyme assay of the supernatant of the control vector (pMt) infected cell with the 20E and Oleoyl-CoA. **(B)** The enzyme assay of the supernatant of the pMt-SATF infected cell with the 20E and Oleoyl-CoA. **(C)** The enzyme assay of the supernatant of the pMt-SATF infected cell with the 20E and water.

In order to examine the enzyme activity of two functional unknown enzymes (Cyp6b2 and SATF), the cotton bollworm genes were expressed in *Drosophila S2* cell. Using western blotting by Flag antibody, one specific band corresponding to the molecular masses of 55 and 60 kDa was detected in the lysis of pMT-SATF and pMT-Cyp6b2 plasmid transfected cells, respectively (Supplementary Figure S5). This result indicated that both genes were successfully expressed in *S2* cell. Thereafter, the crude enzyme solutions were used to measure their activity. As shown in the reversed-phase HPLC UV chromatograms, compared to the control (pMT plasmid transfected cell), crude enzymes from SATF transfected cell can covert 20E into its 22-oleate-acyl ester form (Figure 5). It should be noted that this transformation can only be detected with the supplement of oleoyl-CoA (Figure 5). These data demonstrate that SATF codes for an ecdysteroid-22-O-acyltransferase.

For *cyp6b2* gene, HPLC showed a similar peak profile with control, indicating the enzyme cannot convert 20E (data not shown).

## Discussion

A total of 45% of insect species feed on plants (Schoonhoven et al., 2005). Plants do not just passively tolerate when suffering from phytophagy insects, instead, they evolved some elegant mechanisms to minimize or inhibit the hazard that insects impose on them. 20-hydroxyecdysone, an essential developmental regulator in insects, is also a major phytoecdysteroids in plants. Therefore, the phytoecdysteroid is considered as defense to negatively affect herbivore growth and survival. In parallel, some insects also develop strategies to overcome the defensive system. In this study, we found that *H. armigera* is remarkably tolerate to high concentrations of ingested exogenous 20E (up to 50 μg/larva) without any adverse effects on food consumption and development. Correspondingly, midgut transcriptome analysis also showed most of the digestive enzyme genes kept the same stable expression pattern as the control. This phenomenon maybe resulted from the high efficiency to quickly transform and excrete the ingested 20E within a few hours. In *Heliothis virescens*, Zhang and Kubo (1993) also found over 70% of radioactivity was recovered in feces at 1 h after oral treatment of ^[3 H]^ecdysone (Zhang and Kubo, 1993). Those results indicated some noctuid larvae have a high rate of excretion system to detoxify exogenous hormones.

The midgut is the main tissue for insects to detoxify the plant allelochemicals (Despres et al., 2007). In order to identify the candidate genes contributing to ecdysteroid tolerance of the cotton bollworm, we focused on the 20E induced genes in the midgut transcriptomic data. Several previous studies have demonstrated that some ecdysone degradation pathways, including 3-epimerization and 26-hydroxylation, also were induced by the hormone (Li et al., 2014; Sun et al., 2017). Hence, by comparative transcriptome analysis, we found four genes encoding three cytochrome P450 enzymes (*cyp6b2, cyp18a1*, and *cyp18b1*) and one Long-FACL. Cytochrome P450 enzymes are well known for their roles in the metabolism of insecticide and plant secondary compounds (Heidel-Fischer and Vogel, 2015). Cyp18a1 and Cyp18b1 serve as 26-hydroxylase to convert 20E into 20-hydroxyecdysonoic acid (Rewitz et al., 2010; Guittard et al., 2011). However, we cannot identify their product (20-hydroxyecdysonoic acid) in the cotton bollworm feces. In *H. virescens*, Zhang and Kubo (1993) only detected trace amounts of 20-hydroxyecdysonoic acid in the feces of the larvae orally injected by 20E (Zhang and Kubo, 1993). It should be pointed out that though *Cyp18a1* and *Cyp18b1* genes were highly induced after 20E treatment, our research and previously published papers proved the majority of ingested 20E were converted into acyl-ester forms, indicating the ecdysteroid esterification pathway is the key pathway for detoxifying exogenous hormones (Robinson et al., 1987; Zhang and Kubo, 1993). Hence, the function of the 26-hydroxylases needs further investigation in the future.

In the normal feeding stage of *H. virescens*, the midgut crude enzyme extract showed ecdysteroid-22-*O*-acyltransferase activity only with the supplement of fatty acyl-CoA (Zhang and Kubo, 1992b; Kubo et al., 1994). Those results give us two important hints: (1) the larvae midgut itself contains the acyltransferase under normal conditions and (2) the enzyme activity is fatty acyl-CoA dependent. Indeed, the SATF, which was proven as ecdysteroid-22-*O*-acyltransferase in this study, showed similar expression levels between control and 20E treatment, and the gene was mainly expressed in thr digestive system. In the *H. virescens* midgut, enzyme activity of the acyltransferase also had no change after 20E incubation (Zhang and Kubo, 1992b). However, the transcript level of *Long-FACL* encoding an enzyme to produce fatty acyl-CoA is quite low in the control midgut but can be highly and quickly induced by 20E. As an important regulator, ecdysone firstly binds to the EcR/USP complex and subsequently induces several primary and late response transcription factors (Thummel, 2001). We detected several 20E related cis-regulatory sites, such as EcR/USP, Br-C, and E74, located on the 5′ upstream of the Long-FACL gene indicating it is a 20E inducible gene in the midgut. In total, it would appear that the insect only starts to esterify exogenous ecdysteroid in the presence of acyl esters supplied by a 20E inducible enzyme Long-FACL.

Blackford and Dinan (1997a) proposed that the sensitivity of insects to phytoecdysteroids is correlated to their feeding habits (monophagous insects: sensitive; oligophagous insects: semi-tolerant; polyphagous insects: tolerant) (Blackford and Dinan, 1997a). Our recent results partly explain how a polyphagous insect, the cotton bollworm, utilizes esterification pathway to degrade the exogenous ecdysteroid to conquer the plant defense. However, the genes involved in this pathway are also conserved in lepidopteran insects including *B. mori*, a well-known monophagous and 20E sensitive insect (Kubo et al., 1983). It is an interesting question why domesticated silkworm cannot protect themselves against 20E through the same enzymes as the cotton bollworm. In addition, ecdysteroid-22-acylesters are also found in the ovaries and eggs of some insects, and the hormone derivates are served as storage forms for embryogenesis (Connat et al., 1988; Whiting and Dinan, 1989). Thus, more studies are needed to survey the novel function of the ecdysteroid esterification pathway during insect developmental process in the future.

## Data Availability Statement

The raw data of RNA-seq has been submitted to NCBI (SRA accession: PRJNA588578).

## Ethics Statement

Experiments were conducted in accordance with the protocol approved by the Institutional Animal Care and Use Committee of the Chongqing University (permit number CBE-A201607020).

## Author Contributions

WS and ZZ conceived and designed the experiments. HD, XY, ZB, and XL performed the experiments. HD and XY analyzed the data. ZZ and WS contributed reagents, materials, and analysis tools. HD and WS wrote the manuscript.

## Conflict of Interest

The authors declare that the research was conducted in the absence of any commercial or financial relationships that could be construed as a potential conflict of interest.

## Footnotes

1 http://www.geneontology.org/

2 http://www.genome.jp/kegg/

3 http://www.omicshare.com/tools/

4 http://jaspar.genereg.net/

## References

[B1] Adler J. H. Grebenok R. J. (1999). Occurrence, biosynthesis, and putative role of ecdysteroids in plants. *Crit. Rev. Biochem. Mol. Biol.* 34 253–264. 10.1080/10409239991209282 10517645

[B2] Aly R. Ravid U. Abu-Nassar J. Botnick I. Lebedev G. Gal S. (2011). Biological activity of natural phytoecdysteroids from Ajuga iva against the sweetpotato whitefly *Bemisia tabaci* and the persea mite *Oligonychus perseae*. *Pest. Manag. Sci.* 67 1493–1498. 10.1002/ps.2203 21604353

[B3] Anders S. Pyl P. T. Huber W. (2015). HTSeq-a Python framework to work with high-throughput sequencing data. *Bioinformatics* 31 166–169. 10.1093/bioinformatics/btu638 25260700 PMC4287950

[B4] Blackford M. Clarke B. Dinan L. (1996). Tolerance of the Egyptian cotton leafworm *Spodoptera littoralis* (Lepidoptera: Noctuidae) to ingested phytoecdysteroids. *J. Insect Physiol.* 42 931–936. 10.1016/0022-1910(96)00052-2

[B5] Blackford M. Dinan L. (1997a). The effects of ingested 20-hydroxyecdysone on the larvae of Aglais urticae, Inachis io, Cynthia cardui (Lepidoptera: Nymphalidae) and Tyria jacobaeae (Lepidoptera: Arctiidae). *J. Insect Physiol.* 43 315–327. 10.1016/s0022-1910(96)00112-6 12769893

[B6] Blackford M. Dinan L. (1997b). The effects of ingested ecdysteroid agonists (20-hydroxyecdysone, RH5849 and RH5992) and an ecdysteroid antagonist (cucurbitacin B) on larval development of two polyphagous lepidopterans (*Acherontia atropos* and *Lacanobia oleracea*). *Entomol. Exp. Appl.* 83 263–276. 10.1046/j.1570-7458.1997.00181.x

[B7] Blackford M. J. Clarke B. S. Dinan L. (1997). Distribution and metabolism of exogenous ecdysteroids in the Egyptian cotton leafworm *Spodoptera littoralis* (Lepidoptera: Noctuidae). *Arch. Insect. Biochem. Physiol.* 34 329–346. 10.1002/(sici)1520-6327(1997)34:3<329::aid-arch7>3.0.co;2-p

[B8] Butenandt A. Karlson P. (1954). Über die isolierung eines metamorphose-hormons der insekten in kristallisierter form. *Zeitschrift Naturforschung B* 9 389–391. 10.1515/znb-1954-0601

[B9] Chaubey M. K. (2018). Role of phytoecdysteroids in insect pest management: a review. *J. Agron.* 17 1–10. 10.3923/ja.2018.1.10

[B10] Chen C. Xia R. Chen H. He Y. (2018). TBtools, a Toolkit for Biologists integrating various biological data handling tools with a user-friendly interface. *BioRxiv [Preprint]* 10.1101/289660

[B11] Connat J. L. Dotson E. M. Diehl P. A. (1988). Apolar conjugates of ecdysteroids are not used as a storage form of molting hormone in the argasid tick *Ornithodoros moubata*. *Arch. Insect. Biochem. Physiol.* 9 221–235. 10.1002/arch.940090306

[B12] Despres L. David J.-P. Gallet C. (2007). The evolutionary ecology of insect resistance to plant chemicals. *Trends Ecol. Evol.* 22 298–307. 10.1016/j.tree.2007.02.010 17324485

[B13] Dinan L. (2001). Phytoecdysteroids: biological aspects. *Phytochemistry* 57 325–339. 10.1016/s0031-9422(01)00078-74 11393511

[B14] Götz S. García-Gómez J. M. Terol J. Williams T. D. Nagaraj S. H. Nueda M. J. (2008). High-throughput functional annotation and data mining with the Blast2GO suite. *Nucl Acid Res.* 36 3420–3435. 10.1093/nar/gkn176 18445632 PMC2425479

[B15] Guittard E. Blais C. Maria A. Parvy J.-P. Pasricha S. Lumb C. (2011). CYP18A1, a key enzyme of Drosophila steroid hormone inactivation, is essential for metamorphosis. *Dev. Biol.* 349 35–45. 10.1016/j.ydbio.2010.09.023 20932968

[B16] Heidel-Fischer H. M. Vogel H. (2015). Molecular mechanisms of insect adaptation to plant secondary compounds. *Curr. Opin. Insect Sci.* 8 8–14. 10.1016/j.cois.2015.02.004 32846688

[B17] Hisanaga Y. Ago H. Nakagawa N. Hamada K. Ida K. Yamamoto M. (2004). Structural basis of the substrate-specific two-step catalysis of long chain fatty acyl-CoA synthetase dimer. *J. Biol. Chem.* 279 31717–31726. 10.1074/jbc.m400100200 15145952

[B18] Jurenka R. Russell K. O’Neal M. (2017). Phytoecdysteroids as antifeedants towards several beetles that include polyphagous and monophagous feeding guilds. *Pest. Manag. Sci.* 73 1633–1637. 10.1002/ps.4500 27976533

[B19] Kanehisa M. Goto S. (2000). KEGG: kyoto encyclopedia of genes and genomes. *Nucl Acid Res.* 28 27–30. 10.1093/nar/28.1.27 PMC102409 10592173

[B20] Kang X.-L. Zhang J.-Y. Wang D. Zhao Y.-M. Han X.-L. Wang J.-X. (2019). The steroid hormone 20-hydroxyecdysone binds to dopamine receptor to repress lepidopteran insect feeding and promote pupation. *PLoS Genet.* 15:e1008331. 10.1371/journal.pgen.1008331 31412019 PMC6693746

[B21] Kubo I. Klocke J. A. Asano S. (1981). Insect ecdysis inhibitors from the East African medicinal plant *Ajuga remota* (Labiatae). *Agr. Biol. Chem.* 45 1925–1927. 10.1271/bbb1961.45.1925

[B22] Kubo I. Klocke J. A. Asano S. (1983). Effects of ingested phytoecdysteroids on the growth and development of two lepidopterous larvae. *J. Insect Physiol.* 29 307–316. 10.1016/0022-1910(83)90031-8

[B23] Kubo I. Komatsu S. Asaka Y. de Boer G. (1987). Isolation and identification of apolar metabolites of ingested 20-hydroxyecdysone in frass of Heliothis virescens larvae. *J. Chem. Ecol.* 13 785–794. 10.1007/bf01020160 24302046

[B24] Kubo I. Zhang M. De Boer G. Uchima K. (1994). Location of ecdysteroid 22-O-acyltransferase in the larvae of *Heliothis virescens*. *Entomol. Exp. Appl.* 70 263–272. 10.1111/j.1570-7458.1994.tb00755.x

[B25] Lafont R. Horn D. (1989). “Phytoecdysteroids: structures and occurrence,” in *Ecdysone From Chemistry to Mode of Action*, ed. Koolman J. (New York, NY: Thieme Medical Publishers), 39–64.

[B26] Lam G. T. Jiang C. Thummel C. S. (1997). Coordination of larval and prepupal gene expression by the DHR3 orphan receptor during Drosophila metamorphosis. *Development* 124 1757–1769. 9165123 10.1242/dev.124.9.1757

[B27] Li Z. Ge X. Ling L. Zeng B. Xu J. Aslam A. F. (2014). CYP18A1 regulates tissue-specific steroid hormone inactivation in *Bombyx mori*. *Insect Biochem. Mol. Biol.* 54 33–41. 10.1016/j.ibmb.2014.08.007 25173591 PMC4692384

[B28] Love M. I. Huber W. Anders S. (2014). Moderated estimation of fold change and dispersion for RNA-seq data with DESeq2. *Genome Biol.* 15: 550. 25516281 10.1186/s13059-014-0550-8 PMC4302049

[B29] Martin T. Ochou G. O. Djihinto A. Traore D. Togola M. Vassal J. M. (2005). Controlling an insecticide-resistant bollworm in West Africa. *Agr. Ecosyst. Environ.* 107 409–411. 10.1016/j.agee.2004.11.006

[B30] Mithöfer A. Boland W. (2012). Plant defense against herbivores: chemical aspects. *Annu. Rev. Plant Biol.* 63 431–450. 10.1146/annurev-arplant-042110-103854 22404468

[B31] Mondy N. Caïssa C. Pitoizet N. Delbecque J. P. Corio-Costet M. F. (1997). Effects of the ingestion of *Serratula tinctoria* extracts, a plant containing phytoecdysteroids, on the development of the vineyard pest *Lobesia botrana* (Lepidoptera: Tortricidae). *Arch. Insect. Biochem. Physiol.* 35 227–235. 10.1002/(sici)1520-6327(1997)35:1/2<227::aid-arch21>3.0.co;2-c

[B32] Nakanishi K. Koreeda M. Sasaki S. Chang M. Hsu H. (1966). Insect hormones. The structure of ponasterone A, insect-moulting hormone from the leaves of *Podocarpus nakaii* Hay. *Chem. Comm.* 24, 915–917.

[B33] Pertea M. Kim D. Pertea G. M. Leek J. T. Salzberg S. L. (2016). Transcript-level expression analysis of RNA-seq experiments with HISAT, StringTie and Ballgown. *Nat. Prot.* 11:1650. 10.1038/nprot.2016.095 27560171 PMC5032908

[B34] Rewitz K. F. Yamanaka N. O’Connor M. B. (2010). Steroid hormone inactivation is required during the juvenile-adult transition in Drosophila. *Dev. Cell* 19 895–902. 10.1016/j.devcel.2010.10.021 21145504 PMC3025487

[B35] Robinson P. Morgan E. Wilson I. Lafont R. (1987). The metabolism of ingested and injected [3H] ecdysone by final instar larvae of *Heliothis armigera*. *Physiol. Entomol.* 12 321–330. 10.1111/j.1365-3032.1987.tb00757.x

[B36] Schmelz E. A. Grebenok R. J. Ohnmeiss T. E. Bowers W. S. (2002). Interactions between *Spinacia oleracea* and Bradysia impatiens: a role for phytoecdysteroids. *Arch. Insect. Biochem. Physiol.* 51 204–221. 10.1002/arch.10062 12432520

[B37] Schoonhoven L. M. Van Loon B. van Loon J. J. Dicke M. (2005). *Insect-Plant Biology.* Oxford: Oxford University Press on Demand.

[B38] Soriano I. R. Riley I. T. Potter M. J. Bowers W. S. (2004). Phytoecdysteroids: a novel defense against plant-parasitic nematodes. *J. Chem. Ecol.* 30 1885–1899. 10.1023/b:joec.0000045584.56515.11 15609826

[B39] Sun W. Shen Y. H. Qi D. W. Xiang Z. H. Zhang Z. (2012). Molecular cloning and characterization of Ecdysone oxidase and 3-dehydroecdysone-3α-reductase involved in the ecdysone inactivation pathway of silkworm, *Bombyx mori*. *Int. J. Biol. Sci.* 8:125. 10.7150/ijbs.8.125 22215981 PMC3248655

[B40] Sun W. Wang C. F. Zhang Z. (2017). Transcription factor E74A affects the ecdysone titer by regulating the expression of the EO gene in the silkworm, *Bomby mori*. *Biochim. Biophys. Acta* 1861 551–558. 10.1016/j.bbagen.2016.11.017 27855280

[B41] Tanaka Y. Naya S. (1995). Dietary effect of ecdysone and 20-hydroxyecdysone on larval development of two lepidopteran species. *Appl. Entomol. Zool.* 30 285–294. 10.1303/aez.30.285

[B42] Terra W. R. Ferreira C. (2012). “Biochemistry and molecular biology of digestion,” in *Insect Molecular Biology and Biochemistry*, ed. Gilbert L. I. (London: Elsevier Science Publishers), 365–418. 10.1016/b978-0-12-384747-8.10011-x

[B43] Thummel C. S. (2001). Molecular mechanisms of developmental timing in *C. elegans* and Drosophila. *Dev. Cell* 1 453–465. 10.1016/s1534-5807(01)00060-0 11703937

[B44] Whiting P. Dinan L. (1989). Identification of the endogenous apolar ecdysteroid conjugates present in newly-laid eggs of the house cricket (Acheta domesticus) as 22-long-chain fatty acyl esters of ecdysone. *Insect. Biochem.* 19 759–765. 10.1016/0020-1790(89)90057-7

[B45] Zhang M. Kubo I. (1992a). Characterization of ecdysteroid-22-O-acyltransferase from tobacco budworm, *Heliothis virescens*. *Insect Biochem. Mol. Biol.* 22 599–603. 10.1016/0965-1748(92)90037-f

[B46] Zhang M. Kubo I. (1992b). Possible function of ecdysteroid-22-O-acyltransferase in the larval gut of tobacco budwo*rm. Heliothis virescens*. *J. Chem. Ecol.* 18 1139–1149. 10.1007/bf00980069 24254154

[B47] Zhang M. Kubo I. (1993). Metabolic fate of ecdysteroids in larval *Bombyx mori* and *Heliothis virescens*. *Insect Biochem. Mol. Biol.* 23 831–843. 10.1016/0965-1748(93)90072-z

