# Supplementary material for: Identification and Characterization of Genes Involved in Ecdysteroid Esterification Pathway Contributing to the High 20-Hydroxyecdysone Resistance of Helicoverpa armigera
Source: Front Physiol. 2020 Jun 9;11:508. doi: 10.3389/fphys.2020.00508 (PMC7296158; doi:10.3389/fphys.2020.00508)
Supplement: Supplementary file 1 [file Data_Sheet_1.docx]

**Supporting information**

**Figure legends**

**Supplemental Figure S1. Mass spectrum of the (A) 20-hydroxyecdysone-22-oleate and (B) 20-hydroxyecdysone-22-stearate.**

**Supplemental Figure S2. Metabolic fate of ingested 20E.** (A), HPLC analysis of ecdysteroids extracted from hemolymph of larvae at 6 hours after control or 20E treatment; (B), HPLC analysis of ecdysteroids extracted from fat body of larvae at 6 hours after control or 20E treatment. Black lines represent the extraction from control treated larvae, and red lines represent the extraction from 20E treated larvae.

**Supplemental Figure S3. Phylogeny analysis of insect Cyp18 proteins.** Neighbor-joining method was used to construct phylogeny tree of cyp18 proteins by MEGA X. The *Drosophila melanogaster* phantom which belongs to Cytochromes P450 superfamily was used as the outgroup.

**Supplemental Figure S4. Gene structure and cis-regulatory element prediction of Long-FACL.** (A), gene structure of Long-FACL gene. (B), prediction of cis-regulatory elements in 5' upstream regulatory region (2000 bp) of Long-FACL gene on JASPAR online tool (<http://jaspar.genereg.net/>). The nucleobases with different background represent different cis-regulatory elements.

**Supplemental Figure S5. The western blotting analysis of the heterologous expression for the candidate genes**

**
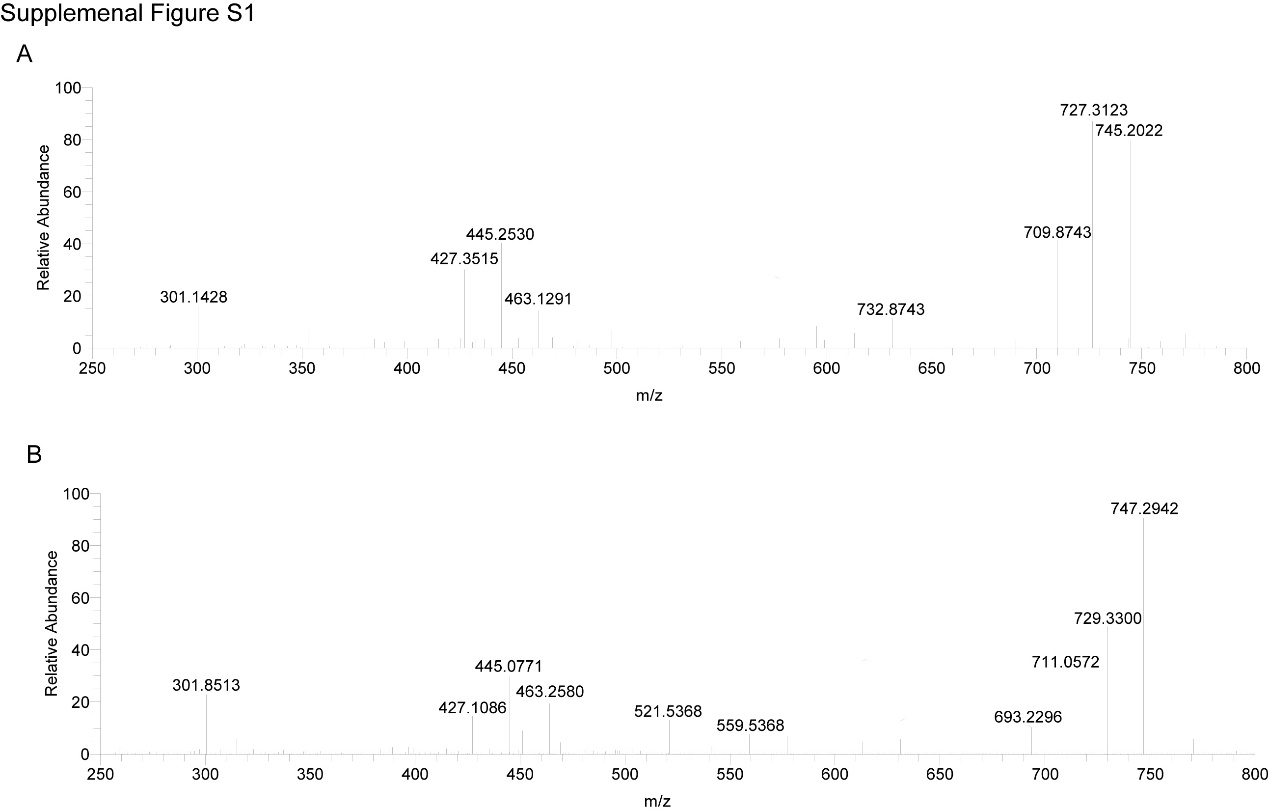
**

**
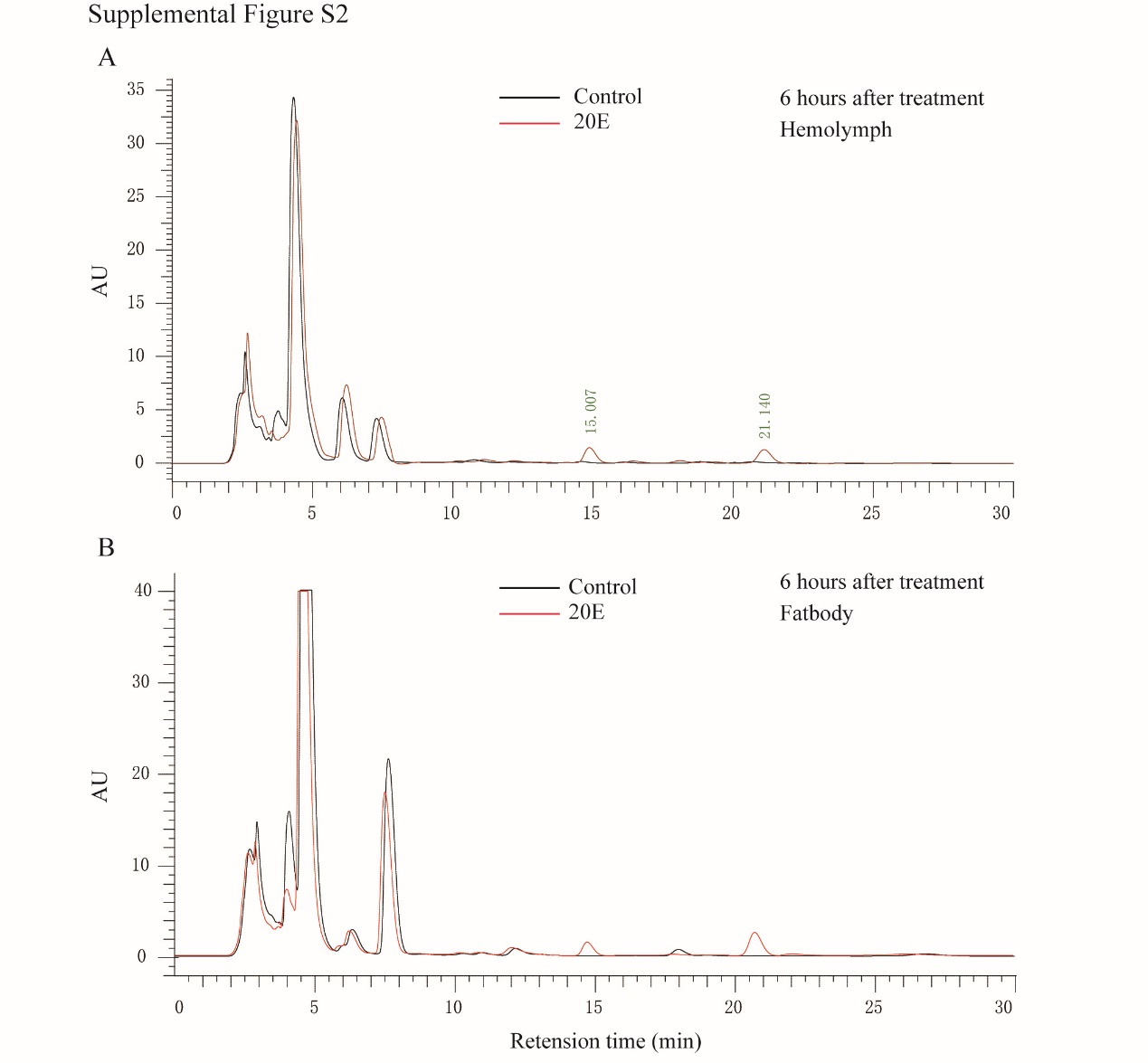

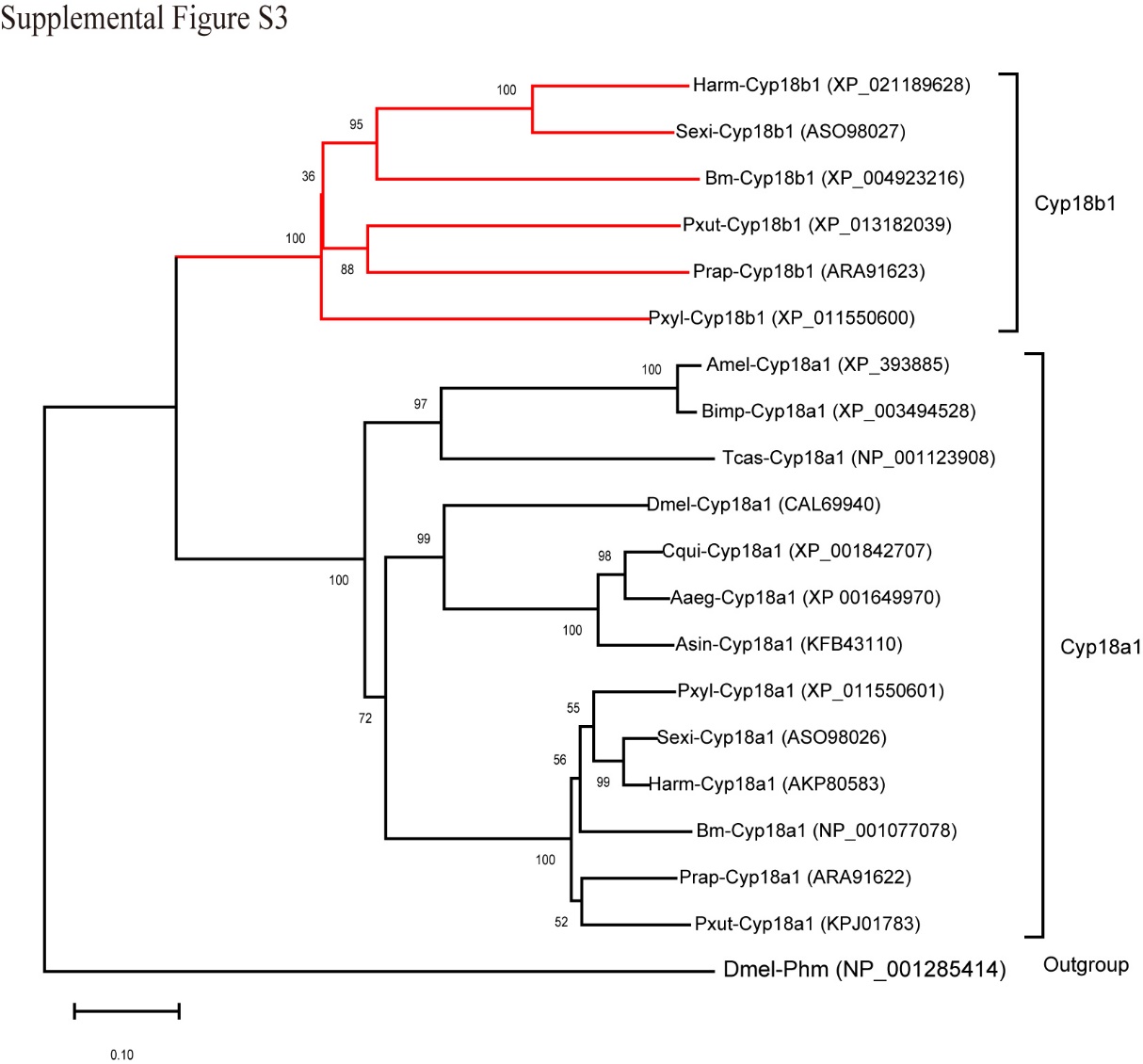

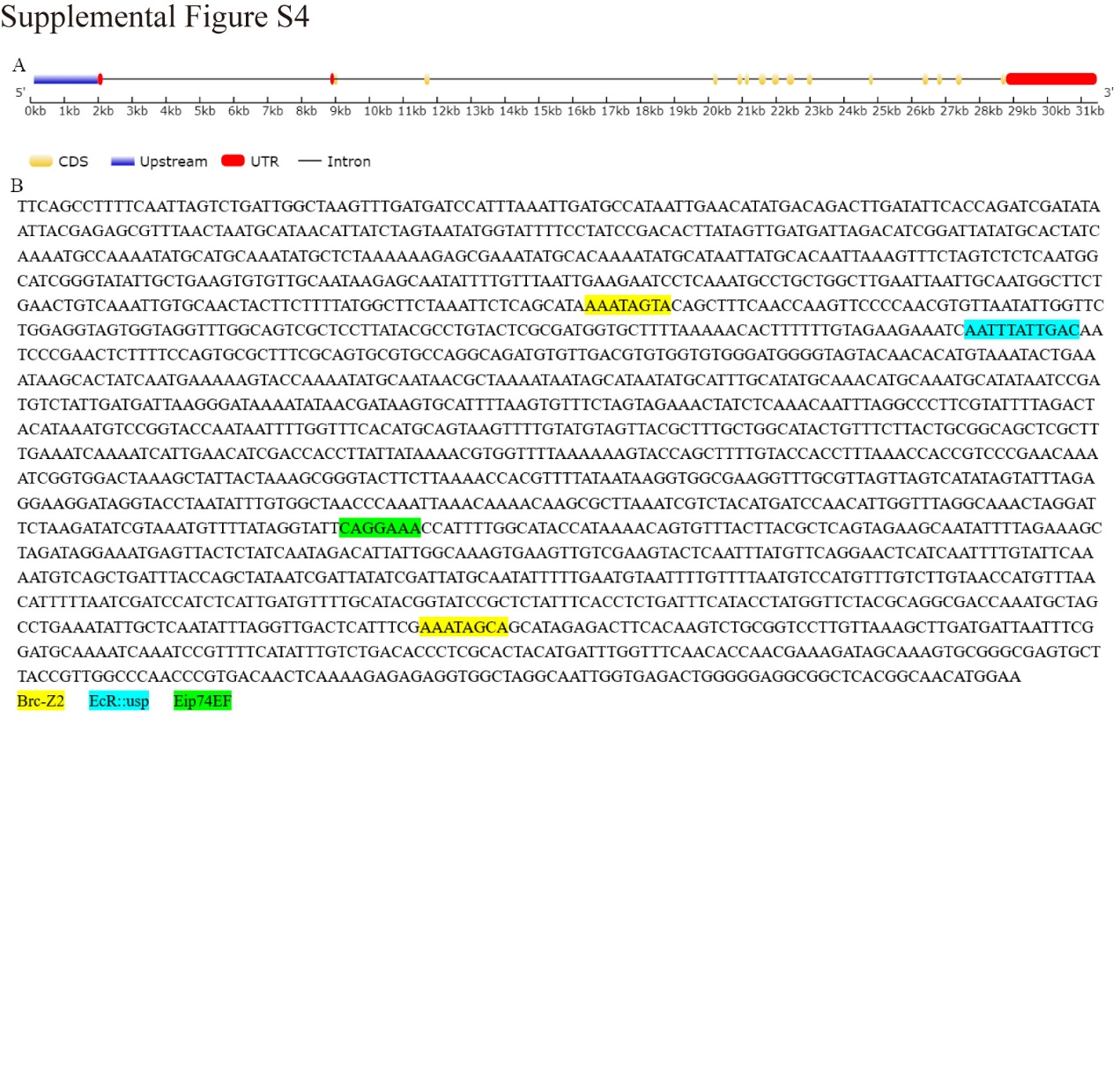

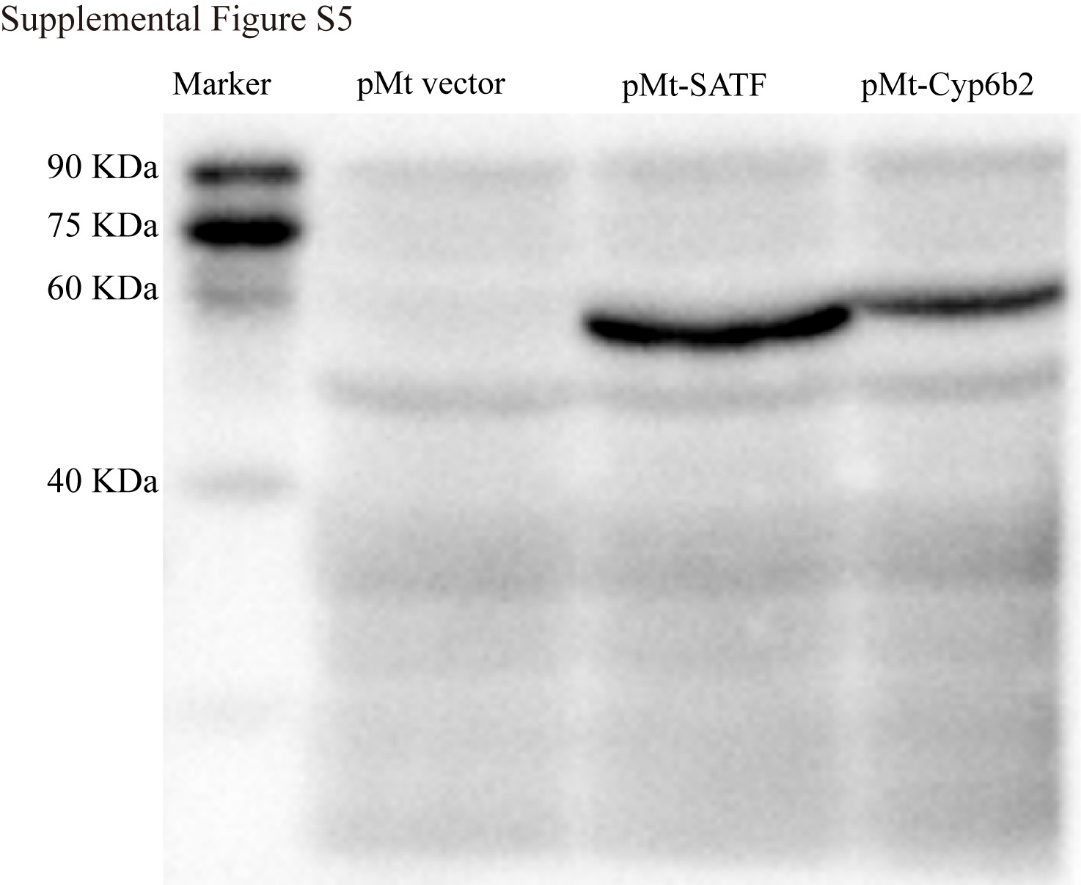
**

**Supplemental Table S1 List of primers used in this study**

| **Usage** | **Primers name** | **Sequence (5’-3’)** |
| --- | --- | --- |
| Real-time PCR | SATF-qRT-F | CCCGACCTTCAAACACTACG |
|  | SATF-qRT-R | TCCTTGCCATAATCAGACCA |
|  | Long-FACL-qRT-F | GCCATCCACGCATCAAAGAG |
|  | Long-FACL-qRT-R | GCCGAGTTGCATGATGACGCT |
|  | Cyp18a1-qRT-F | CGCTCATGCATACTTTGGAT |
|  | Cyp18a1-qRT-R | TGGCGATAAGCTCGTACACT |
|  | Cyp18b1-qRT-F | AACGAAAGGGTGTCCAGAGG |
|  | Cyp18b1-qRT-R | GGTGAAACTGGCTCAGGTCC |
|  | Cyp6B2-qRT-F | CCCGGAACGGTTCTATGGAG |
|  | Cyp6B2-qRT-R | GCTCTGAGCCTTGAACGTGA |
|  | RPL3-qRT-F | TCTTCCAAGTTCGGTCACGG |
|  | RPL3-qRT-R | GGATGCGGTCCTTCTTGAGT |
| Heterologous expression | SATF-pMT-F | CACGCTAGCATGGGCAAAATGAAGGAAAACGG |
|  | SATF-pMT-R | CACGCGGCCGCTTAGTTTGTCAAAATACGTAAAGGA |
|  | Cyp6B2-pMT-F | CACGCTAGCATGTTCTTCTACTTACTCGTG |
|  | Cyp6B2-pMT-R | CACGCGGCCGCTCACTTCACAAAAACTCTGTTCTCC |

**Supplemental Table S2 Identification and expression of genes encoding transcription factors.**

| **Gene ID** | **AVG. TPM-Control** | **AVG. TPM-20E** | **log2(fc)** | **FDR** | **Annotation** |
| --- | --- | --- | --- | --- | --- |
| MSTRG.6353 ¶ | 0.07 | 743.26 | 13.37422 | 7.3E-272 | HR3 |
| MSTRG.7078 ¶ | 0.69 | 44.67 | 6.016566 | 1.03E-23 | HR4 |
| MSTRG.9351 | 0.235 | 5.215 | 4.471935 | 0.015186 | DNA-binding protein D-ETS-6 |
| MSTRG.11537 ¶ | 0.485 | 6.505 | 3.745492 | 0.014399 | E93 |
| MSTRG.7501 | 1.01 | 12.335 | 3.610331 | 7.41E-05 | CCAAT/enhancer-binding protein |
| MSTRG.9408 ¶ | 3.185 | 30.125 | 3.241596 | 7.91E-11 | Krueppel homolog 1-like |
| MSTRG.8770 | 21.61 | 174.8 | 3.015934 | 3.46E-51 | transcription factor Sox-12 |
| MSTRG.1313 ¶ | 2.16 | 13.92 | 2.688056 | 0.000232 | Broad-Complex |
| MSTRG.2752 ¶ | 46.9 | 300.14 | 2.677976 | 1.8E-72 | E75 |
| MSTRG.2806 ¶ | 20.52 | 119.13 | 2.537434 | 5.36E-30 | EcR |
| MSTRG.7595 | 43.79 | 142.14 | 1.698639 | 9E-21 | nuclear factor interleukin-3-regulated protein |
| MSTRG.13202 | 36.755 | 106.085 | 1.529208 | 1.33E-13 | Steroid hormone receptor ERR1 |
| MSTRG.640 | 30.845 | 67.155 | 1.122458 | 5.67E-06 | Forkhead box protein O |
| MSTRG.11804 | 36.155 | 78.435 | 1.117302 | 7.95E-07 | HMG box transcription factor |
| MSTRG.10687 | 75.19 | 157.945 | 1.07081 | 2.79E-11 | Zinc finger protein |

¶ represents the 20E responsible transcription factors

**Supplemental Table S3 Identification and expression of genes encoding membrane-bound O-acyltransferase (MBOAT)**

| **Gene ID** | **AVG. TPM-Control** | **AVG. TPM-20E** | **log2(fc)** | **FDR** | **Annotation** | **Predicted subcellular localization** |
| --- | --- | --- | --- | --- | --- | --- |
| MSTRG.4506 | 82.785 | 63.855 | -0.37457 | 0.0555223 | Diacylglycerol O-acyltransferase | Plasma membrane |
| MSTRG.6506 | 63.98 | 44.11 | -0.53652 | 0.1922225 | Lysophospholipid acyltransferase | Endoplasmic reticulum |
| MSTRG.7066 | 0.7 | 1.095 | 0.5073416 | 1 | Protein-serine O-palmitoleoyltransferase porcupine | Plasma membrane |
| MSTRG.8235 | 20.44 | 20.67 | 0.016143 | 1 | Sterol O-acyltransferase | Plasma membrane |
